# Evaluation of the Individual Effects of Melatonin and Umbilical Cord-Derived Mesenchymal Stem Cell Exosomes on Cell Viability and Apoptosis in BE(2)-C Neuroblastoma Cells In Vitro

**DOI:** 10.3390/cimb48060623

**Published:** 2026-06-16

**Authors:** Ahmet Şengül, Dilek Kaan, Hatice Güler, Hüseyin Yiğit

**Affiliations:** 1 Institution of Health Sciences, Erciyes University, Kayseri 38039, Turkey; asengul2538@gmail.com; 2 Betul Ziya Eren Genome and Stem Cell Center, Erciyes University, Kayseri 38039, Turkey; 3 Halil Bayraktar Vocational Health College, Erciyes University, Kayseri 38039, Turkey; 4 Department of Anatomy, Faculty of Medicine, Erciyes University, Kayseri 38039, Turkey; hsusar@erciyes.edu.tr; 5 Department of Medical Services and Techniques, Vocational Health School, Cappadocia University, Nevşehir 50420, Turkey; anatomisth@gmail.com

**Keywords:** BE(2)-C, exosome, melatonin, mesenchymal stem cell, neuroblastoma

## Abstract

The study aimed to investigate the individual therapeutic effects of melatonin and umbilical cord-derived mesenchymal stem cell exosomes (UC-MSC-Exo) separately on BE(2)-C neuroblastoma cells. Melatonin is recognized for its anti-cancer, antioxidant, and apoptosis-inducing properties, and its ability to cross the blood–brain barrier. UC-MSC-Exos are nanovesicles from mesenchymal stem cells that can also cross the blood–brain barrier and transport biologically active molecules. The potential therapeutic benefits of each independent agent in treating BE(2)-C neuroblastoma cells were investigated. Melatonin and UC-MSC-Exos were examined on BE(2)-C neuroblastoma cells at varying concentrations and time intervals to evaluate cell viability and apoptosis. Both melatonin and UC-MSC-Exo independently reduced cell viability and induced apoptosis in a manner that depended on the dosage and duration of exposure. Melatonin had an IC_50_ of 2.68 mM after 24 h, while UC-MSC-Exo showed an IC_50_ of 25.3 μg/mL after 48 h, with no cytotoxic effects observed at 24 h. Specifically, individual concentrations of 2.5 mM and 5 mM of melatonin, as well as 50 µg/mL and 100 µg/mL of UC-MSC-Exo, led to significant levels of apoptotic and necrotic cells at 48 and 72 h (*p* < 0.001). Our findings suggest that the individual administration of melatonin and UC-MSC-Exo may hold therapeutic potential for neuroblastoma cells, particularly given their ability to cross the blood–brain barrier. Further in vivo research is required to evaluate their clinical utility.

## 1. Introduction

Neuroblastoma, the second most common solid tumor of the central nervous system, arises from developmental abnormalities in the embryonic neural crest [1], typically occurring in the adrenal medulla, paraspinal region, and sympathetic ganglia [2]. This cancer predominantly affects children aged 0 to 6 years, with metastasis detected in about 50% of cases [3].

Treatment strategies for neuroblastoma are individualized based on the patient’s risk assessment, involving a sequence of induction chemotherapy, primary surgery, stem cell transplantation, and radiation therapy [1]. However, conventional treatments may result in severe side effects like organ damage, anemia, fertility issues, and hair loss. These standard approaches have limited efficacy against advanced stages of the disease and do not effectively target minimal residual disease, increasing the risk of potential relapses. The complex nature of neuroblastoma, its immune evasion mechanisms, and cellular heterogeneity present significant challenges for existing therapies such as chemotherapy, surgery, and radiation [4]. Therefore, the development of novel therapeutic strategies is crucial for managing this disease.

Exosomes, small vesicles released from cells, are essential for intercellular communication by transporting various biomolecules [5,6]. They are present in bodily fluids like urine, blood, breast milk, and cerebrospinal fluid, and can also be released by mesenchymal stem cells (MSCs) [7]. Research has shown that MSC-exosomes (MSC-exo) containing specific microRNAs can enhance brain recovery and regeneration following injury [8,9,10,11]. Unlike MSCs, exosomes can easily traverse the blood–brain barrier, making them a promising therapeutic option for neurological injuries. MSC-exos can reduce prolonged inflammation, aid tissue healing, maintain tissue stability, mitigate heart damage due to reduced blood flow, guard against kidney injury, facilitate wound and inflammation recovery, and assist injured cells in absorbing therapeutic proteins and RNAs [12]. This extends their therapeutic impact from cell-to-cell interactions to broader paracrine interactions [13].

Melatonin, a hormone produced by the pineal gland, readily crosses the blood–brain barrier. Known as the body’s internal clock, melatonin plays a role in regulating circadian rhythms, modulating the immune system, reducing inflammation, acting as an antioxidant, regulating blood vessels, and potentially inhibiting cancer growth [14]. Studies have found decreased levels of melatonin in various neurological disorders like stroke, Alzheimer’s, and Parkinson’s. Fortunately, patients treated with melatonin therapy have shown improvements in their condition [5]. Many researchers believe that melatonin’s exceptional antioxidant properties make it a critical factor in the development of neurological conditions such as epileptic seizures, stroke, brain damage, and neurotrauma, as well as neurodegenerative diseases like Alzheimer’s disease and Parkinsonism, due to oxidative stress being a primary contributor [15].

This study investigates the potential for developing distinct baseline strategies in neuroblastoma treatment, as both agents have the individual ability to cross the blood–brain barrier and offer potentially complementary apoptotic mechanisms. In this study, we aimed to investigate the standalone effects of melatonin and exosomes derived from human umbilical cord tissue mesenchymal stem cells on the BE(2)-C neuroblastoma cell line separately. Our hypothesis is that these substances will inhibit the growth of high-risk neuroblastoma cells and reduce their survival rates.

## 2. Materials and Methods

The cellular procedures in this study were conducted in compliance with ethical guidelines. The Erciyes University Clinical Research Ethics Committee waived the requirement for ethical approval for this in vitro study on 19 January 2022 (Decision No: 56).

### 2.1. Umbilical Cord-Derived Mesenchymal Stem Cells

Umbilical cord-derived mesenchymal stem cells (UC-MSCs) were received frozen from the Betül-Ziya Eren Genome and Stem Cell Center (GENKOK) and then thawed. They were cultured in DMEM complex medium with 15% FBS (Cat#A5670701, Thermo FisherScientific, Waltham, MA, USA), 1% penicillin/streptomycin, and 2 mM L-glutamine at 37 °C with 5% CO_2_ for 48 h. During the final 48 h, the cells were cultured without FBS [16]. UC-MSCs were analyzed by flow cytometry. Cells were incubated with antibodies against CD73, CD90, CD105, CD44 and hematopoietic markers (negative control). Analysis was performed using a BD FACS Aria III flow cytometer(BD Biosciences, San Jose, CA, USA). UC-MSCs were positive for CD73, CD90, CD105 and CD44 (>95%). They were negative hematopoietic markers for CD34/CD45/CD11b/CD19/HLA-DR (<2%).

### 2.2. Cell Culture of the BE(2)-C Neuroblastoma Cell Line

The Human Neuroblastoma BE(2)-C cell line (ATCC #CRL-2268, isolated from the brain of a male patient with neuroblastoma) was grown in a 1:1 mixture of DMEM/F12 and RPMI-1640 media, supplemented with 10% FBS, 1% antibiotic solution, and 1% glutamine. The cells were then placed in a 5% CO_2_ incubator and incubated at 37 °C for 48 h.

### 2.3. Exosome (UC-MSC-Exo) Isolation and Characterization from Umbilical Cord-Derived Mesenchymal Stem Cells

Exosomes were extracted from MSCs cultured in FBS-free medium for 48 h using the ExoQuick-TC kit from System BioSciences (Cat. #EXOTC50A-1, System BioSciences, Palo Alto, CA, USA). Initially, 20 mL of the FBS-free cell culture supernatant was collected and centrifuged at 3000× *g* for 15 min to eliminate cellular debris (HIMAC, CP100NX- P55STN2, Hitachi Koki Co., Ltd., Hitachinaka, Japan). Then, 1/5 volume of exosome precipitation solution was added to the supernatant and mixed well by vortexing. The mixture was left to incubate at +4 °C for 24 h. Following the incubation, the exosomes were collected by centrifugation at 1500× *g* for 30 min. Exosomes morphology was examined using Field Emission Scanning Electron Microscopy (FESEM) for morphology, and their diameter was determined using ImageJ software(version 1.54) [17]. Exosome stability and suitability for our study were evaluated by measuring the zeta potential with a ZetaSizer device (Nano ZS, Malvern Panalytical, Malvern, UK) [18]. Nanoparticle tracking analysis (NTA) was conducted using a Nanosight NS300 system (Malvern Panalytical, Malvern, UK) to determine particle size distribution and concentration. This technique utilizes light scattering and Brownian motion principles to analyze nanoparticles in suspension [19]. The exosome suspensions were incubated with PE- and APC-conjugated antibodies against CD63 and CD81 at 4 °C for 30 min in the dark. Subsequently, the samples were washed using 0.22 µm-filtered PBS and centrifuged at 20,000× *g* for 30 min at 4 °C to remove any unbound antibodies. The pellet was resuspended in PBS and analyzed on a FACS Aria III flow cytometer. Instrument settings were optimized for particles under 200 nm via calibration with 100 nm and 200-nm beads. Isotype controls were utilized to eliminate non-specific background noise. Data were processed using FACS Diva software (version 8.0), where gating strategies were applied to exclude debris and aggregates, enabling the quantification of exosomes based on their CD63 and CD81 positivity.

### 2.4. Cytotoxicity Analysis

Cell viability was evaluated using the XTT kit according to the manufacturer’s instructions (Luminex, Austin, TX, USA), (Glowmax Elisa Raeder, Promega, Madison, WI, USA). BE(2)-C neuroblastoma cells were plated at a density of 10^4^ cells per well in a 96-well plate. After 24 h, the cells were switched to serum-free medium for another 24 h. Subsequently, the cells were treated with different concentrations of melatonin (1, 2.5, and 5 mM) or UC-MSC-exo (25, 50, and 100 µg/mL) separately for 24, 48, and 72 h. Cell viability was determined by measuring absorbance at 450 nm using a spectrophotometer. Prior to initiation of the main experiments, a preliminary dose-finding assessment was conducted to identify a non-cytotoxic concentration range of melatonin in BE(2)-C cells; thus, the selected treatment doses were chosen based on a safety threshold that preserves basal cell viability while enabling mechanistic investigation of apoptotic and cytotoxic endpoints. Melatonin demonstrates significant biological effects in various tumor cell lines including neuroblastoma at pharmacological concentrations (≈0.5–2 mM). In SH-SY5Y neuroblastoma cells, 1 mM melatonin functionally reduces VEGF-mediated tumor-related angiogenesis [20]. Moreover, in human gastric cancer SGC7901 cells, ~2 mM melatonin induced pronounced apoptosis after 24 h [21]. This literature justifies the use of the 1–5 mM dose range in our study as a high-dose pharmacological, mechanistic-screening threshold rather than a physiological one, supporting the selection of the mM range to determine the ultimate cytotoxic boundaries and overcome the high baseline resistance typically observed in aggressive, high-risk neuroblastoma lines during initial in vitro screenings [22].

### 2.5. Annexin-FITC/Dead Cell Double Staining

The level of apoptosis in BE(2)-C cells post-treatment with melatonin and exosomes was assessed using the Muse Annexin V & Dead Cell Assay kit (Luminex Corporation, Austin, TX, USA). This kit utilizes Annexin V to detect the externalization of phosphatidylserine (PS) and phosphatidylethanolamine (PE) molecules on the cell surface, indicating early signs of apoptosis or cell death. The kit also includes 7-AAD dye to stain dead cells, enabling the quantification of non-viable cells. BE(2)-C cells were seeded at a density of 1 × 10^6^ cells/well in 6-well plates with 1 mL of medium per well and incubated for 24 h at 37 °C in a 5% CO_2_ incubator. After the initial 24-h period, the medium was changed to FBS-free complex medium. Following an additional 24 h of incubation in serum-free medium, cells were treated with different concentrations of melatonin (1, 2.5, and 5 mM) or UC-MSC-exo (25, 50, and 100 µg/mL) separately for 24, 48, and 72 h. Apoptosis and cell death were then assessed using a BD FACS Aria III flow cytometer device.

### 2.6. Statistical Analysis

The data from the apoptosis method was analyzed using GraphPad Prism 8, two-way ANOVA, and Tukey’s multiple comparisons test. Cytotoxicity and apoptosis data were obtained from at least three independent biological replicates (performed on different cell culture passages) and analyzed using GraphPad Prism 8 with a significance value of *p* < 0.05. All experiments were performed in triplicate and repeated at least three times independently.

## 3. Results

### 3.1. Characterization of UC-MSCs and BE(2)-C Cell Line

**Figure 1:**
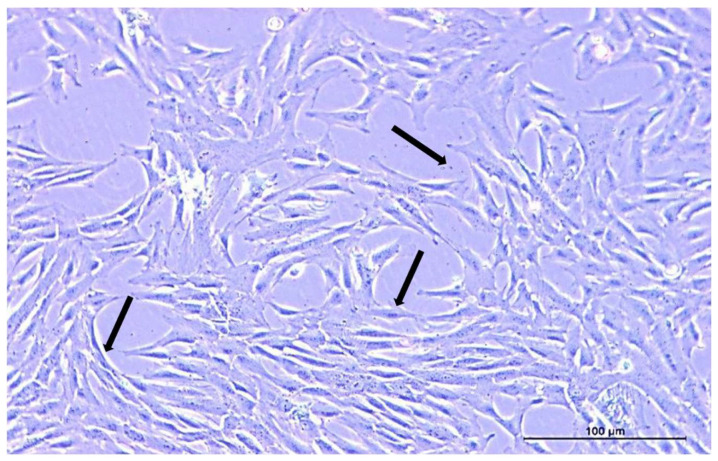
Light microscopy image of mesenchymal stem cells (MSCs) cultured in vitro showing typical spindle-shaped morphology. Arrows indicate representative spindle-shaped MSCs characterized by elongated cell bodies with tapering ends and centrally located oval nuclei. Scale bar = 100 µm.

**Figure 2:**
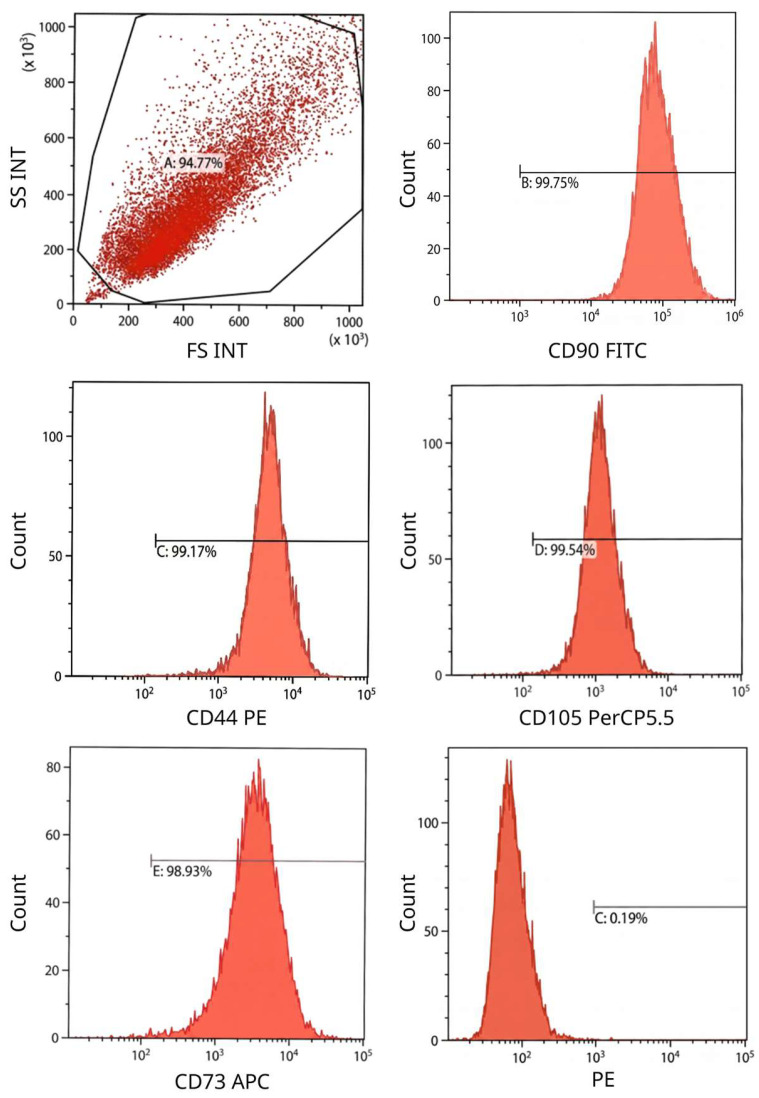
Flow cytometric analysis of umbilical cord-derived mesenchymal stem cells (UC-MSCs). Cells were stained with antibodies against MSC-specific surface markers CD73, CD90, CD105, and CD44, along with a hematopoietic negative control. Forward and side scatter plot showing the initial gating strategy used to exclude debris and select the viable cell population (94.77%). Histogram plots demonstrating high expression of MSC-positive markers CD90 (99.75%), CD44 (99.17%), CD105 (99.54%), and CD73 (98.93%). Histogram plot representing the negative control (PE), showing negligible signal (0.1%). This confirms the absence of hematopoietic contamination and validates the specificity of the positive marker expression.

**Figure 3:**
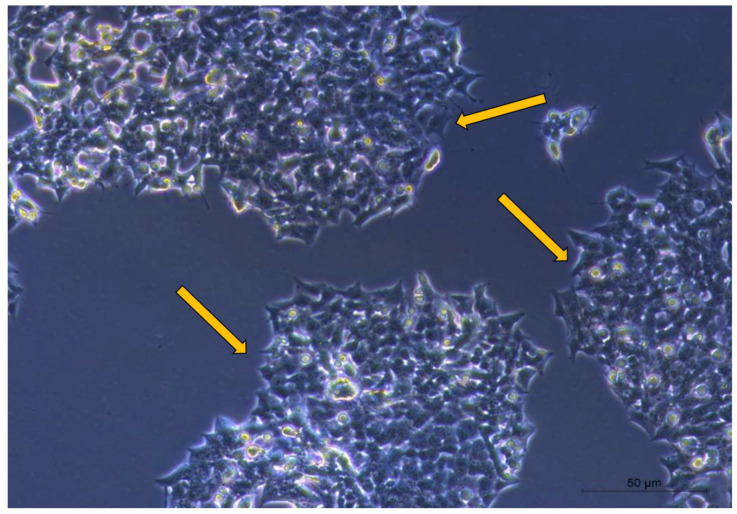
Light microscopy image of BE(2)-C neuroblastoma cells. Arrows indicate round or polygonal-shaped cells with distinct nucleus–cytoplasm boundaries, exhibiting a typical epithelioid morphology and a tendency to form clustered arrangements. Scale bar = 50 µm.

On the fourth day, MSCs were examined for adherence and morphology. By day eight, the MSCs displayed their characteristic spindle-shaped appearance, achieved 80–90% confluence, and remained firmly attached to the flask base (Figure 1). Flow cytometric analysis confirmed the mesenchymal phenotype of UC-MSCs. Cells exhibited high expression of CD90 (99.75%), CD44 (99.17%), CD105 (99.54%), and CD73 (98.93%) In contrast, expression of hematopoietic markers CD34/CD45/CD11b/CD19/HLA-DR were negligible (0.1%). The homogeneous distribution of marker expression and the high positivity rates indicate a pure and well-characterized MSC population. These results confirm that the isolated cells meet the minimal criteria for mesenchymal stem cells (Figure 2). These well-characterized UC-MSCs were subsequently used for exosome isolation. BE(2)-C neuroblastoma cells (ATCC, CRL-2268) exhibited typical adherent growth and were observed under an inverted microscope (Figure 3).

### 3.2. Exosome Isolation and Characterization

**Figure 4:**
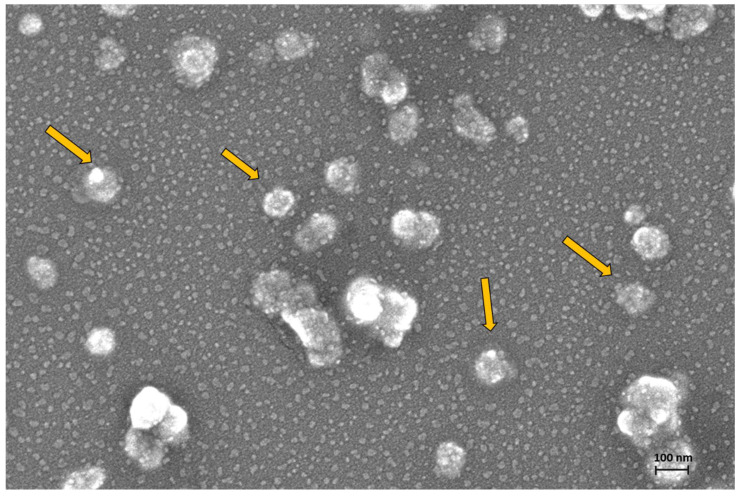
Scanning Electron Microscopy (SEM) image of UC-MSC-derived exosomes. Arrows indicate spherical, membrane-bound nanovesicles with smooth surfaces and diameters typically ranging between 80–120 nm, consistent with the characteristic morphology of exosomes. Scale bar = 100 nm.

The isolated extracellular vesicles displayed the characteristic morphology of exosomes. Under scanning electron microscopy (SEM), spherical, membrane-enclosed nanovesicles were uniformly distributed, with a mean diameter of 101.63 ± 3.1 nm measured via ImageJ, falling precisely within the typical exosome size range (Figure 4). Flow cytometric analysis complemented these morphological findings, confirming their identity through key tetraspanin markers CD63 and CD81. Specifically, 37.8% of the gated particles exhibited expression of CD63, while 67.2% showed CD81 positivity. The stronger CD81 signal points to potential molecular heterogeneity within the exosome population, which is frequently documented for those derived from mesenchymal stem cells (MSCs). Our gating strategy effectively excluded cellular debris and aggregates, isolating distinct single vesicle events (Supplementary Figure S1). Zeta potential measurements revealed a surface charge of −13.3 mV, indicating favorable colloidal stability and resistance to aggregation, confirming that the exosomes remained stable in suspension. The distinct single-peak profile reinforced the uniform composition of the vesicle preparation (Supplementary Figure S2). Nanoparticle tracking analysis (NTA) provided further quantification of vesicle size and concentration, yielding a mean of 87.9 nm and a concentration of 3.421 × 10^8^ particles/mL. The size distribution curve peaked strictly within established exosome boundaries, and the 3D intensity plot demonstrated steady uniformity, underscoring the high purity of the preparation (Supplementary Figures S3 and S4).

### 3.3. Cytotoxicity Assay

**Figure 5:**
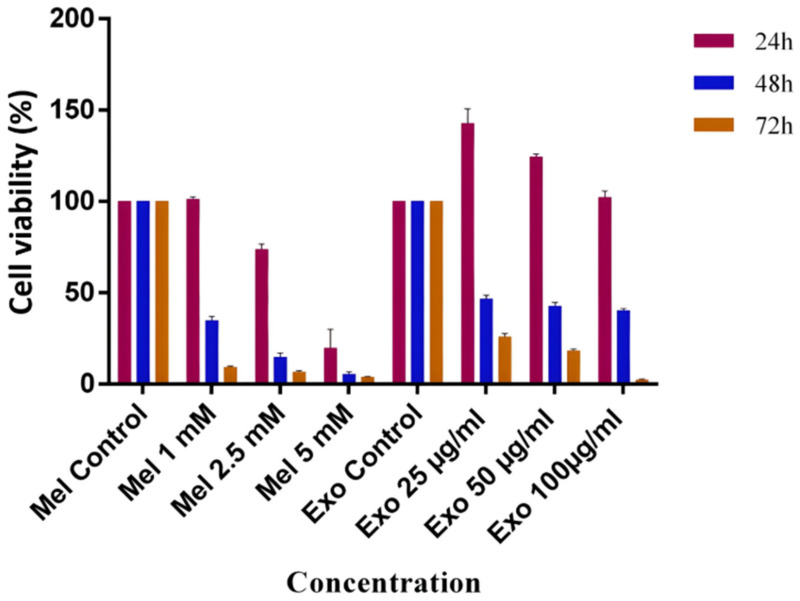
Effect of melatonin and exosomes on percentage of cell viability at 24, 48 and 72 h in BE(2)-C cells. The results are presented as mean ± SD of three independent biological replicates.

Table 1 shows the XTT method results of the individual cytotoxic effects of UC-MSC-Exo and melatonin on cell viability based on concentration and time. The comparative result graph is displayed in Figure 5. The IC_50_ of melatonin was calculated as 2.68 mM for 24 h, 1.41 mM for 48 h, and 1.089 mM for 72 h (Supplementary Figure S5). For exosomes, the IC_50_ was calculated as 25.3 μg/mL for 48 h. No IC50 could be determined for 24 h as exosomes increased cell viability at this time point. Statistical analysis revealed significant differences in percentage cell viability over time for both independent treatment groups compared to the control group and between treatments (*p* = 0.0085). Similarly, % cell viability showed significant differences based on concentration when comparing the control group and the concentration group (*p* = 0.0091). While exosomes increased viability at 24 h, they showed cytotoxic effects at 48 and 72 h.

### 3.4. Apoptosis Analysis Results via Annexin-FITC/Dead Cell Double Staining

**Figure 6:**
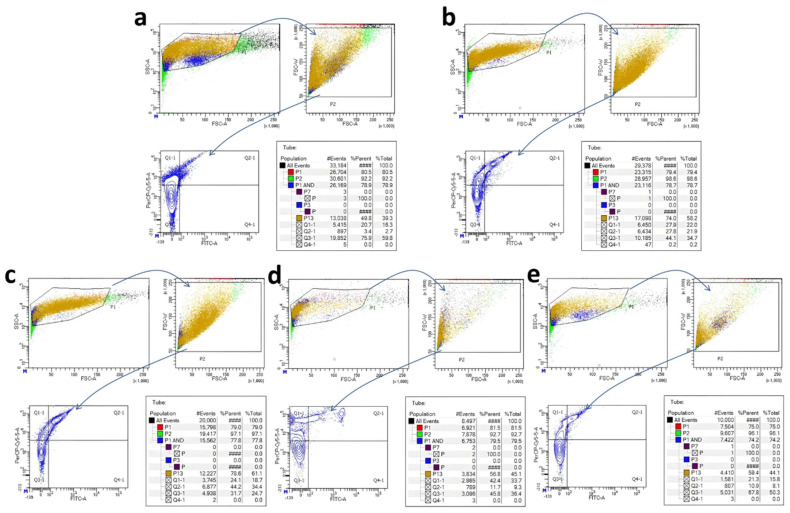
Effect of melatonin and exosome on apoptosis rates at 48 h on BE(2)-C cells. (**a**) Control group. (**b**) 2.5 mM melatonin, (**c**) 5 mM melatonin, (**d**) 50 µg/mL exosome, and (**e**) 100 µg/mL exosome treated groups are shown with Annexin V/PI analysis to show apoptotic cell distribution. Debris was removed by FSC/SSC gating. Based on Annexin V/PI distribution, cells were classified as viable (Q4), early apoptotic (Q3), late apoptotic (Q2), and necrotic (Q1). The graphs are representative of three independent biological replicates.

**Figure 7:**
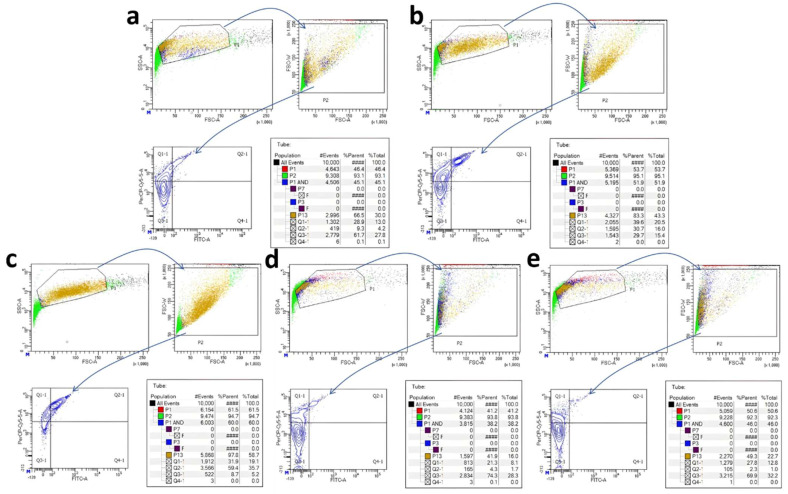
Effect of melatonin and exosome on apoptosis rates at 72 h on BE(2)-C cells. (**a**) Control group. (**b**) 2.5 mM melatonin, (**c**) 5 mM melatonin, (**d**) 50 µg/mL exosome, and (**e**) 100 µg/mL exosome treated groups are shown with Annexin V/PI analysis to show apoptotic cell distribution. Debris was removed by FSC/SSC gating. Based on Annexin V/PI distribution, cells were classified as viable (Q4), early apoptotic (Q3), late apoptotic (Q2), and necrotic (Q1). The graphs are representative of three independent biological replicates.

Table 2 shows that no significant apoptotic induction was observed in BE(2)-C cells at different concentrations of either standalone melatonin or exosome treatments after 24 h. However, at 48 and 72 h, the apoptosis values of both individual melatonin and exosome groups in BE(2)-C cells showed statistically significant changes compared to the control group (** *p* < 0.001). The percentages of viable cells, early-stage apoptotic cells, late-stage apoptotic cells, and necrotic cells were determined using the Annexin V & 7-AAD method. It was found that melatonin and exosomes independently induced apoptosis in BE(2)-C cells at different concentrations and time intervals. The flow cytometry results of apoptosis values following separate treatments with melatonin **or** exosome at 48 h and 72 h in BE(2)-C cells are shown in Figure 6 and Figure 7.

## 4. Discussion

Neuroblastoma arises from the sympathetic nervous system ganglia due to developmental anomalies of the embryonic neural crest, which are pluripotent cells emerging from the dorsal aspect of the neural tube during the third week of embryogenesis. The disease presents with a wide spectrum, from tumors that regress spontaneously to treatment-resistant tumors with metastatic spread despite aggressive therapy [23].

Melatonin, a hormone produced by the pineal gland, plays a role in regulating sleep–wake cycles, controlling blood pressure, and promoting brown adipose tissue formation. It also acts as a powerful antioxidant by binding to melatonin receptors MT1 and MT2. Additionally, melatonin has been shown to protect against neuronal cell death signals caused by endoplasmic reticulum stress, which is linked to age-related disorders like type 2 diabetes, obesity, and insulin resistance. A study found that melatonin can reduce these cell death signals in response to insulin stimulation [24].

Melatonin inhibits liver cancers by reducing cellular proliferation, inducing cell cycle arrest, restricting angiogenesis and metastasis, and promoting cellular death through various molecular and cellular processes [25]. For instance, research conducted on CCA liver cancer cell lines demonstrated these impacts when exposed to concentrations of 0.5, 1, and 2 mM for a duration of 48 h [26]. Additionally, studies have demonstrated that melatonin can enhance the pro-apoptotic effects of cisplatin, a common antitumor drug. In laboratory tests, the combination of cisplatin and 1 mM melatonin increased intrinsic pathway apoptosis in Hep3B, HCC, and Bel-7402 cells, improving treatment outcomes by blocking the NF-kB/COX-2 pathways [27]. In our study, we assessed the therapeutic effects of melatonin on the BE(2)-C neuroblastoma cell line by measuring cell viability with the XTT method. We found a significant decrease in cell viability, down to 4.15% at a 5 mM concentration after 48 h. Additionally, when examining the apoptotic effects of melatonin using the Annexin V method, we observed statistically significant changes in apoptosis values for each concentration at both the 48-h and 72-h time points, compared to the control group (** *p* < 0.001). Specifically, after 48 h, the percentage of late-stage apoptotic cells increased to 44.2% at a 5 mM melatonin concentration. While we fully concur with the Reviewer regarding the translational limitations inherent to high millimolar concentrations (1–5 mM), such supraphysiological ranges are widely established in preliminary in vitro oncology screenings to identify maximum efficacy thresholds and clarify downstream apoptotic signaling before moving onto complex drug carriers. It is important to acknowledge that these concentrations are purely pharmacological and may not be readily achievable systemically in patients using standard clinical formulations. Therefore, the present findings should be interpreted strictly as mechanistic and hypothesis-generating. Future in vivo studies using clinically relevant exposure levels, advanced nanocarrier-mediated targeted encapsulation, or alternative delivery strategies are needed to bridge this translational gap safely.

MSCs are stem cells that are multipotent and originate from the mesoderm during embryogenesis [28]. UC-MSCs have advantages over bone marrow-derived MSCs due to their high proliferative capacity, versatile differentiation potential, and low immunogenicity. UC-MSCs express various markers and exhibit potent immunomodulatory activity, making them attractive for therapeutic applications such as inhibiting tumor growth, improving outcomes post-hematopoietic stem cell transplantation, and promoting hematopoiesis in hematological diseases [29]. Flow cytometric analysis of umbilical cord-derived mesenchymal stem cells (UC-MSCs). Cells were stained with antibodies to MSC-specific surface markers CD73, CD90, CD105, and CD44, and a hematopoietic negative control was also used. The initial gating strategy applied for debris removal and selection of the viable cell population is shown (94.77%). Histograms show high expression levels of MSC-specific markers: CD90 (99.75%), CD44 (99.17%), CD105 (99.54%), and CD73 (98.93%). The PE histogram representing the negative control shows a negligible signal level (0.1%). This confirms the absence of hematopoietic contamination and the specificity of the positive markers. High positivity rates and low background signal, when considered together, indicate that the cell population exhibits homogeneous and well-characterized mesenchymal stem cell characteristics.

The relationship between MSCs and cancer is marked by an intricate, ever-changing equilibrium. MSCs have the ability to selectively travel to tumor locations, potentially aiding in tumor development. On the other hand, cancer cells could be attracted by MSCs located in the bone marrow, resulting in bone metastasis. Importantly, MSCs are known for creating a supportive environment for tumors, promoting tumor growth and spread [30]. MSCs can also contribute to tumor progression by modulating the cancer microenvironment, facilitating neovascularization, secreting different growth factors, and inducing immunosuppression [31]. Moreover, MSCs play a role in cancer metastasis through the secretion of pro-metastatic soluble factors like chemokines such as SDF-1, IL-6, and CCL5 [32].

To circumvent the functional limitations and potential tumor-promoting risks associated with intact MSC transplantation, utilizing cell-free extracellular vesicles (EVs)—specifically MSC-derived exosomes—has emerged as a highly effective paradigm [33,34]. While raw MSC therapies frequently suffer from low systemic biodistribution, infusion toxicities, and potential cellular rejection, MSC-exos retain the key immunomodulatory and regenerative properties of their parent cells with a significantly lower immunogenic and tumorigenic footprint [34]. For instance, bioengineered EVs modified to express therapeutic ligands like TRAIL have shown potent antitumor efficacy in murine models, confirming that these nanovesicles can successfully carry specialized anti-cancer signaling without the biological hazards of direct stem cell grafting [33].

MSC-exos have gained attention for treating severe steroid-resistant asthma because of their strong immunomodulatory effects. A study showed that giving umbilical cord-derived MSC-exos to mice with this condition reduced TRAF1 expression and influenced NF-kB and PI3K/AKT signaling pathways by encouraging macrophage M2 polarization [35].

MSC-derived exosomes have shown positive effects in various animal models of liver disease, such as drug-induced acute liver injury, liver fibrosis, and hepatocellular carcinoma. Their smaller size, simpler structure compared to MSCs, and ease of production and storage provide unique benefits. For example, in a study by Lou et al., human umbilical cord MSC-derived exosomes were used in a carbon tetrachloride (CCl4)-induced liver injury model in Kunming mice. The exosomes were found to improve liver fibrosis by inhibiting epithelial–mesenchymal transition and collagen production by hepatocytes. They also restored serum aspartate aminotransferase activity and suppressed the TGF-β1/Smad signaling pathway by reducing collagen type I/III levels and TGF-β1 and Smad2 phosphorylation [36].

MSC-derived exosomes are highly effective therapeutic vehicles due to their rich content of biologically active molecules, particularly proteins and RNAs. A significant portion of the proteins in exosomes are enzymes, which have catalytic activities and are influenced by their microenvironment. This enzyme-centric nature of exosomes may help reduce the risk of dosing errors when used as therapeutic agents. Notably, MSC-exos contain a cluster of glycolytic enzymes that could potentially improve glycolytic function and ATP production in reperfused myocardium if used therapeutically [37].

Neuroblastoma evades immune system control and shows significant cellular heterogeneity, limiting the effectiveness of current intensive approaches. High-risk neuroblastoma, in particular, has resistance even to immunotherapy, indicating a complex biological landscape that traditional treatments struggle to overcome [38]. The need for new, more effective, and less toxic therapeutic agents for neuroblastoma is urgent due to severe side effects, limited efficacy in advanced stages, and challenges with minimal residual disease. Melatonin has been shown in meta-analyses to significantly improve remission rates (RR = 1.98, 95% CI 1.52 to 2.58) and one-year survival rates (RR = 1.90, 95% CI 1.28 to 2.83) when used as an adjuvant therapy in cancer patients [39]. Another meta-analysis also supported these findings, showing a significantly higher tumor remission rate (RR = 2.25; 95% CI, 1.86–2.71; *p* < 0.00001) and a substantial increase in overall survival (RR = 2.07; 95% CI, 1.55–2.76; *p* < 0.00001) in the melatonin group compared to control groups. The benefits were particularly notable in patients with non-small cell lung cancer (RR = 2.13) and various other solid tumors (RR = 2.31) [40]. Melatonin’s ability to easily cross the blood–brain barrier is a major advantage in treating neuroblastoma, a condition often involving central nervous system components. This allows melatonin to work directly within the central nervous system, bypassing the challenge that other treatments face in crossing this protective barrier [41].

MSCs-exos are seen as potential candidates for cell therapy in different diseases, such as cancer. One example is TRAIL-engineered exosomes showing effectiveness against tumors in melanoma models. These exosomes are currently being studied for their potential as drug carriers for various conditions, including tumors [42]. Importantly, exosomes derived from MSCs have the ability to enter circulation, reach distant targets, and successfully penetrate the blood–brain barrier. This allows them to be absorbed by neurons and glial cells [43]. Moreover, UC-MSC-Exos have demonstrated the ability to decrease microglial pyroptosis caused by oxygen–glucose deprivation/reperfusion and mitigate neuronal damage in a neonatal hypoxic–ischemic brain injury model in vitro, suggesting neuroprotective properties [42].

Our research shows that both melatonin and UC-MSC-Exo can independently induce apoptosis in BE(2)-C neuroblastoma cells. Melatonin activates the intrinsic apoptotic pathway and suppresses pro-survival pathways like NF-κB/COX-2 [27]. UC-MSC-Exos, as nanovesicles carrying biologically active molecules, can deliver standalone pro-apoptotic signals directly to target cells by crossing the blood–brain barrier. While our current experiments evaluated these agents strictly in isolation, their distinct mechanisms suggest a theoretical rationale for future co-treatment strategies. Speculatively, combining these two agents in future studies might yield additive therapeutic effects by simultaneously enhancing mitochondrial dysfunction and suppressing anti-apoptotic signals. Such a future multifaceted approach could potentially increase therapeutic efficacy by targeting different survival mechanisms of neuroblastoma cells, a hypothesis that remains to be validated empirically.

In our study, significant differences were observed in the therapeutic effects of UC-MSC-Exos in BE(2)-C cells when evaluated with the XTT method, as well as in the cytotoxic effects among the groups and within themselves (* *p* < 0.05). The apoptotic effects, evaluated with the annexin V method, showed statistically significant changes in apoptosis values at different concentrations compared to the control group (** *p* < 0.001) when analyzed according to the 48 and 72 h time groups.

While this study establishes a valuable foundational comparison between the independent anti-cancer properties of melatonin and UC-MSC-Exo in vitro, we recognize that these findings represent an initial exploratory stage. Neuroblastoma is a highly heterogeneous disease, and expanding this comparative validation to a broader panel of genetically diverse cell lines will be beneficial to fully generalize these outcomes. Furthermore, while our current scope focused strictly on evaluating baseline independent dose-responses, characterizing the detailed downstream molecular signaling pathways (such as specific oxidative stress markers, mitochondrial membrane potentials, and autophagic flux) via Western blotting and Confocal Microscopy remains an important next step. Similarly, formal combination analyses (e.g., using the Chou–Talalay method) will be essential to accurately evaluate potential synergistic interactions between these two agents. Finally, because the millimolar concentrations of melatonin utilized here serve as pharmacological screening thresholds rather than physiological references, exploring targeted delivery strategies or advanced in vivo formulations will be vital to bridge the translational gap from bench to potential clinical concepts.

This study provides an important contribution to the current literature by investigating the separate therapeutic potentials of melatonin and umbilical cord-derived mesenchymal stem cell exosomes on BE(2)-C neuroblastoma cells in vitro. It supports the existing literature highlighting the urgent need for new therapeutic agents in neuroblastoma treatment due to the severe side effects of standard approaches, their limited efficacy in advanced stages, and their inability to address minimal residual disease [44]. The study’s findings show that both melatonin and UC-MSC-Exo significantly reduce cell viability and induce apoptosis in a concentration and time dependent manner. This represents a significant advantage for neuroblastoma, a tumor involving components of the central nervous system, especially considering the ability of both agents to cross the blood–brain barrier.

## 5. Conclusions

The current work should be considered as hypothesis-generating and exploratory. Clinical translation will require additional in vitro and in vivo validation, more comprehensive mechanistic work, and careful consideration of dosing and delivery strategies. The independent baseline data established for these two agents may pave the way for designing a more effective and multifaceted future combination treatment plan for neuroblastoma. Further in vivo studies are needed to establish appropriate treatment protocols and validate these promising results.

## Figures and Tables

**Table 1:** Percentage of cell viability value of melatonin and UC-MSC-Exo in BE(2)-C cells.

BE(2)-C% Cell Viability					
	Concentration	Control	24 h	48 h	72 h
Melatonin	1 mM	%100	%102.60	%32.30	%8.50
	2.5 mM	%100	%70.40	%12.50	%6.50
	5 mM	%100	%27.40	%4.15	%3.70
UC-MSC-EXO	25 µg/mL	%100	%151.10	%44.50	%27.60
	50 µg/mL	%100	%126.30	%41.10	%18.60
	100 µg/mL	%100	%105.92	%40.80	%1.80

**Table 2:** Percentage apoptosis values of melatonin and UC-MSC-Exo in BE(2)-C cells.

BE(2)-C 48 h	Control	2.5 mM Mel	5 mM Mel	50 µg/mL Exo	100 µg/mL Exo
Necrotic Cells	20.7	27.9	24.1	42.4	21.3
Late-Stage Apoptotic Cells	3.4	27.8	44.2	11.7	10.9
Viable Cells	75.9	44.1	31.7	45.8	67.8
Early-Stage Apoptotic Cells	0	0	0	0.0	0
BE(2)-C 72 h	Control	2.5 mM Mel	5 mM Mel	50 µg/mL Exo	100 µg/mL Exo
Necrotic Cells	28.9	39.6	31.9	21.3	27.8
Late-Stage Apoptotic Cells	9.3	30.7	59.4	4.3	2.3
Viable Cells	61.7	29.7	8.7	74.3	69.9
Early-Stage Apoptotic Cells	0.1	0	0	0.1	0

## Data Availability

The datasets used and/or analyzed during the current study are available from the corresponding authors on reasonable request.

## Supplementary Materials

The following supporting information can be downloaded at: https://www.mdpi.com/article/10.3390/cimb48060623/s1.

## Abbreviations

BE(2)-C, human neuroblastoma cell line; IC_50_, half-maximal inhibitory concentration; NTA, nanoparticle tracking analysis; UC-MSC, umbilical cord-derived mesenchymal stem cell; UC-MSC-Exo, umbilical cord-derived mesenchymal stem cell-derived exosome; XTT, 2,3-bis-(2-methoxy-4-nitro-5-sulfophenyl)-2H-tetrazolium-5-carboxanilide.
