# Supplementary material for: Evaluation of the Individual Effects of Melatonin and Umbilical Cord-Derived Mesenchymal Stem Cell Exosomes on Cell Viability and Apoptosis in BE(2)-C Neuroblastoma Cells In Vitro
Source: Curr Issues Mol Biol. 2026 Jun 16;48(6):623. doi: 10.3390/cimb48060623 (PMC13297956; doi:10.3390/cimb48060623)
Supplement: Supplementary file 1 [file cimb-48-00623-s001.zip › cimb-4355222-supplementary.pdf]

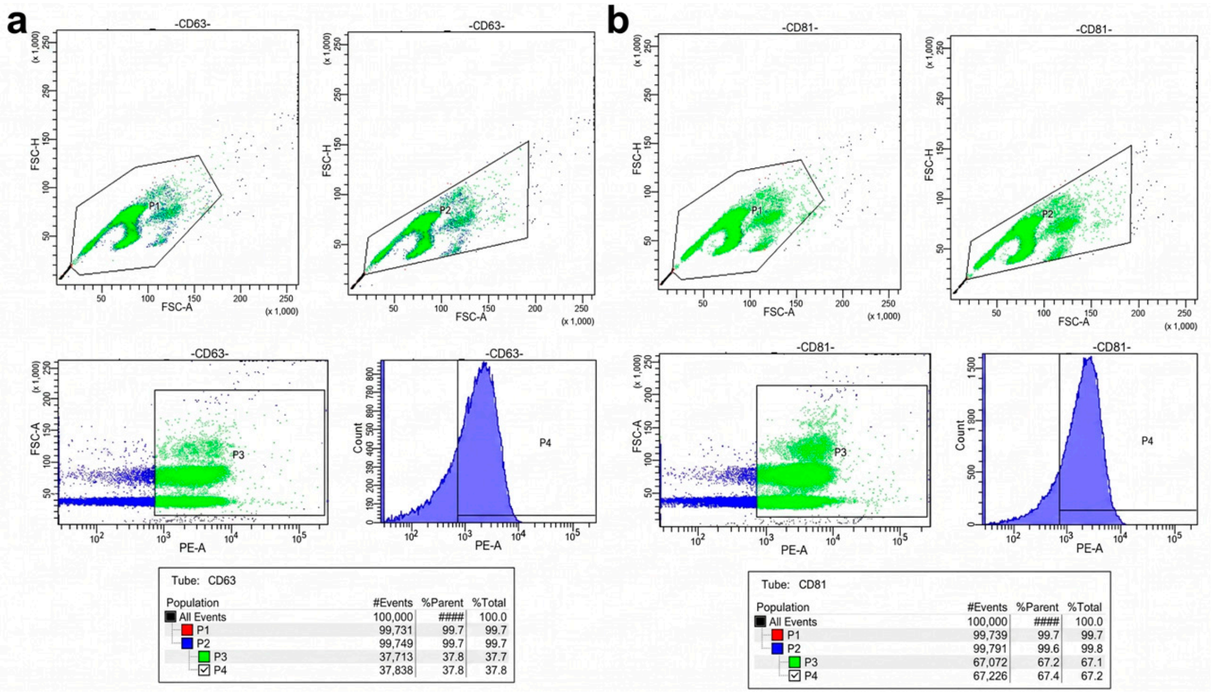

Supplementary Figure S1. Flow cytometric analysis of exosomes for CD63 (**a**) and CD81 (**b**) surface markers was performed using a bead-based method. Exosomes were attached to latex beads and stained with fluorescent conjugated antibodies specific to CD63 and CD81. FSC-A and FSC-H plots show the gating strategy applied to exclude debris and non-specific particles (P1 and P2). Dot plots show the distribution of CD63 and CD81-positive events within the gated population (P3). Histogram analysis reveals the distribution of fluorescence intensity for marker expression (P4). Quantitative analysis revealed CD63 positivity at 37.8% and CD81 positivity at 67.2%. Non-specific bindings were eliminated using isotype controls.

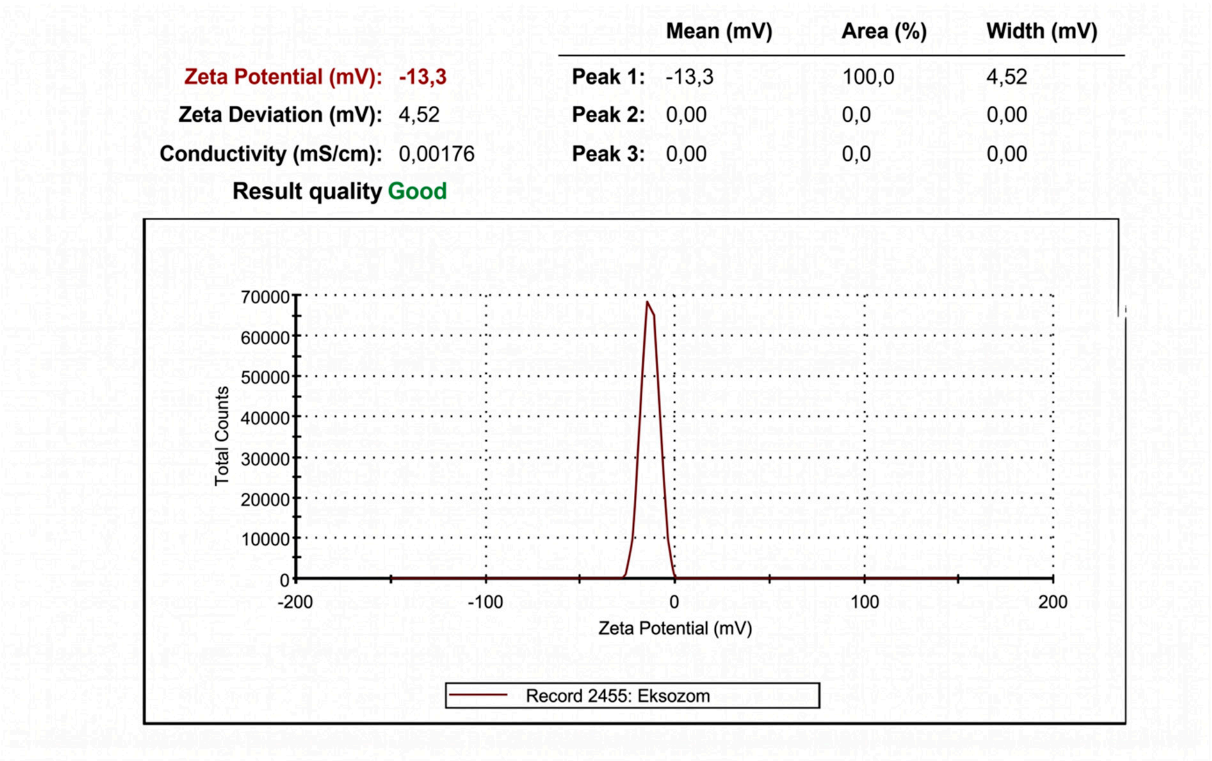

Supplementary Figure S2. The zeta potential of the exosomes was determined to be −13.3 mV. The single-peak distribution indicates that the sample is homogeneous and moderately stable.

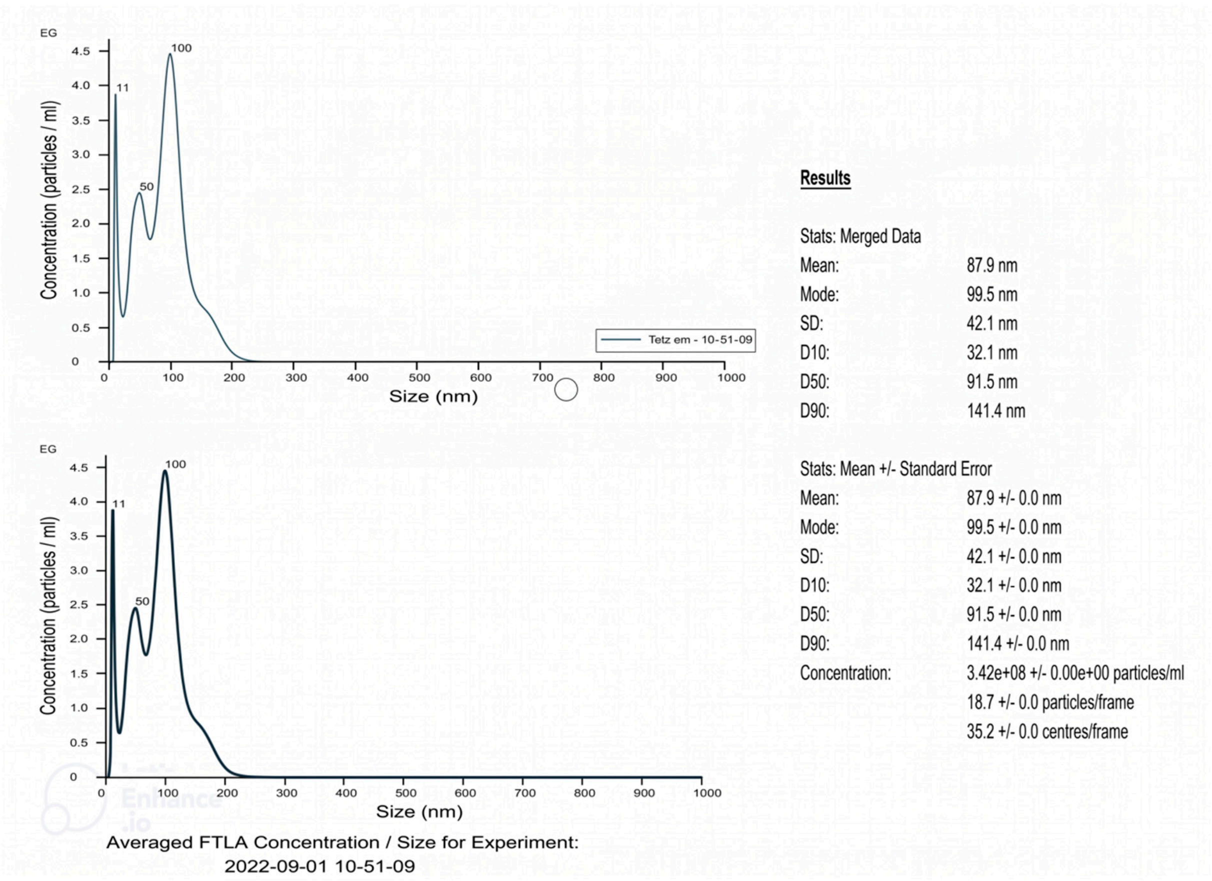

Supplementary Figure S3. Size distribution and concentration of exosomes by NTA. The average particle size was 87.9 nm, and the concentration was 3.42×10^8 particles/mL. The distribution graph shows that the exosomes are concentrated within the expected size range.

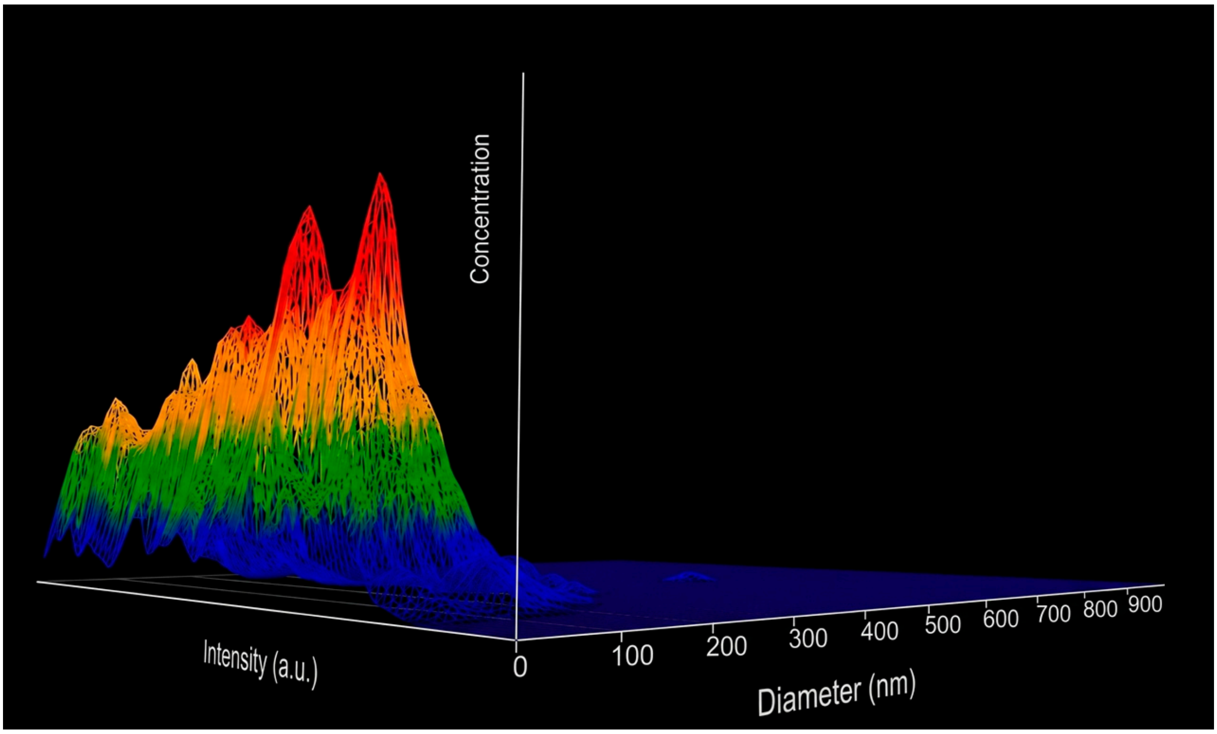

Supplementary Figure S4. Three-dimension concentration and size distribution plot of exosomes with NTA. The size and density distributions of exosomes were analyzed using three-dimensional methods. The graph reveals that the particles are concentrated within specific size ranges and, for example, exhibit a homogeneous distribution.

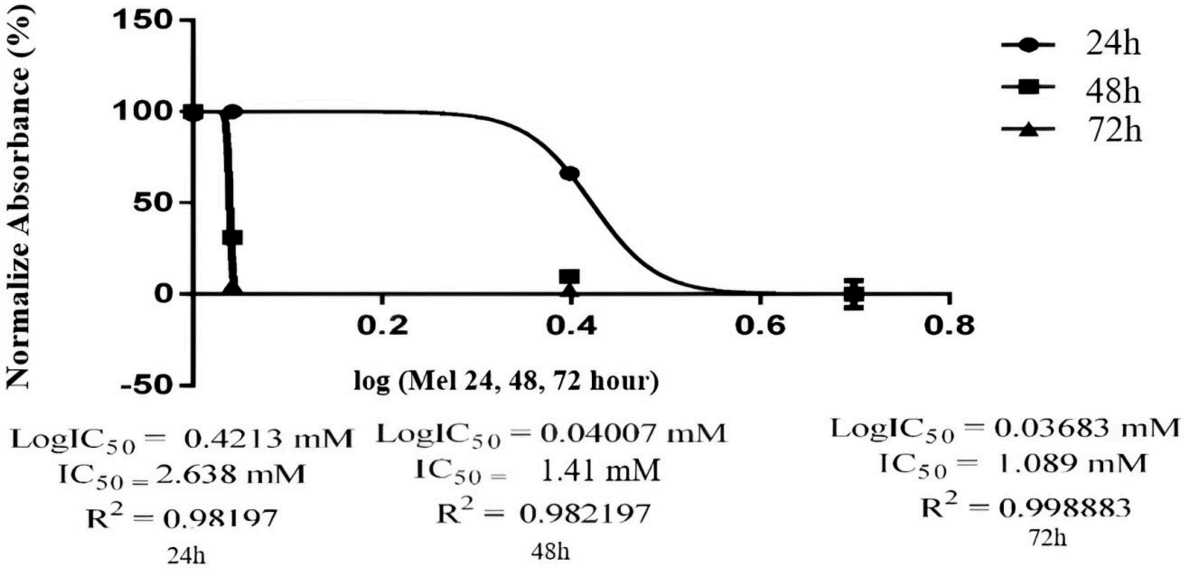

Supplementary Figure S5. The effects of melatonin administration on cell viability are shown at 24, 48, and 72 hours. It was determined that IC_{50} values decreased over time and that melatonin exhibited a time-dependent increase in cytotoxicity.
